# Glucosinolate Content in Dormant and Germinating *Arabidopsis thaliana* Seeds Is Affected by Non-Functional Alleles of Classical Myrosinase and Nitrile-Specifier Protein Genes

**DOI:** 10.3389/fpls.2019.01549

**Published:** 2019-11-26

**Authors:** Kathrin Meier, Markus D. Ehbrecht, Ute Wittstock

**Affiliations:** Institute of Pharmaceutical Biology, Technische Universität Braunschweig, Braunschweig, Germany

**Keywords:** glucosinolates, turnover, germination, seedling, myrosinase, nitrile-specifier protein

## Abstract

While the defensive function of glucosinolates is well established, their possible role as a nutrient reservoir is poorly understood and glucosinolate turnover pathways have not been elucidated. Previous research showed that glucosinolate content in germinating seeds of *Arabidopsis thaliana* Columbia-0 (Col-0) increases within the first two to four days on culture medium and then decreases below the level at day 0. In this study we used previously characterized T-DNA mutants to investigate if enzymes known to be involved in glucosinolate breakdown upon tissue damage affect the time course of glucosinolate content in germinating seeds. Besides dormant seeds, we analyzed seeds subjected to stratification in water for up to 72 h or germination on plates for up to ten days. Although seeds of *tgg1 tgg2* (deficient in above-ground classical myrosinases) had higher glucosinolate levels than Col-0, the changes during germination were not different to those in seeds of Col-0. This demonstrates that TGG1/TGG2 are not responsible for the decline in glucosinolate content upon germination and suggests the involvement of other enzymes. Expression data extracted from publically available databases show a number of β-glucosidases of the BGLU18–BGLU33 clade to be expressed at specific time points of seed maturation and germination identifying them as good candidates for a role in glucosinolate turnover. Although nitrile-specifier proteins (NSPs) act downstream of myrosinases upon glucosinolate breakdown in tissue homogenates, mutants deficient in either seed-expressed NSP2 or seedling-expressed NSP1 were affected in glucosinolate content in seeds and during stratification or germination when compared to Col-0 indicating a direct role in turnover. The mutant lines *nsp1-1*, *nsp2-1* and *nsp2-2* had significantly higher glucosinolate levels in dry seeds than Col-0. After 24 h of stratification in water, *nsp2-2* seeds contained 2.3 fold higher levels of glucosinolate than Col-0 seeds. This might indicate downregulation of hydrolytic enzymes when nitrile formation following glucosinolate hydrolysis is impaired. The time course of total glucosinolate content during ten days of germination depended on functional *NSP1*. Based on the present data, we propose a number of experiments that might aid in establishing the pathway(s) of glucosinolate turnover in germinating *A. thaliana* seeds.

## Introduction

A dual role of specialized metabolites as defenses and nutrient deposits has been discussed for more than 50 years due to the frequent observation that specialized metabolite levels decrease upon germination, maturation or nutrient constraints and more recent reports on growth defects in mutants with reduced levels of specialized metabolites. For example, transgenic cassava lines with RNAi supression of the cyanogenic glycoside biosynthetic pathway did not produce roots *in vitro*, but root growth was restored upon supplementation with nitrogen (Jørgensen et al., 2005). Only in few cases have turnover pathways been elucidated at the molecular level (Rosenthal, 1992; Negi et al., 2014). In addition to serving plant nutrition, turnover of stored specialized metabolites can also contribute to regulation of specialized metabolite levels as in case of flower color changes upon bud opening (Zipor et al., 2015) or diurnal variation of glucosinolate content (Huseby et al., 2013). Thus, “turnover” in the context of specialized metabolism can generally be described as catabolic reactions that yield building blocks (e.g. ammonia, pyruvate, amino acids) for primary metabolism, e.g. under nutrient deficient conditions, or regulate specialized metabolite levels. Although a clear distinction might not always be possible, this differentiates “turnover” from other catabolic reactions, namely “activation” (associated with a gain in biological activity and roles in organismic interactions or signalling) and “detoxification” (conversion to less harmful compounds).

**Figure 1:**
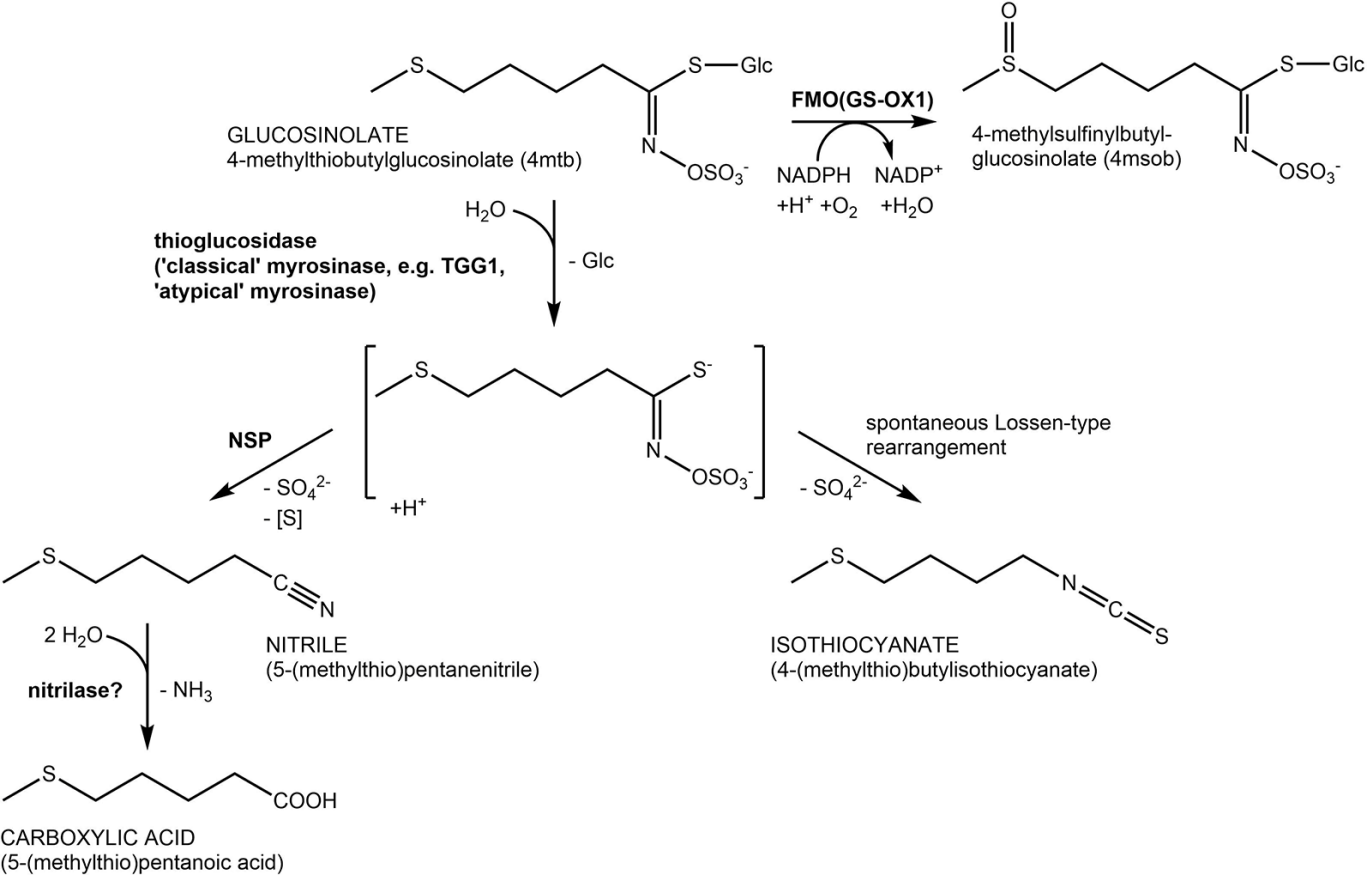
Glucosinolate structures and breakdown pathways. Reactions are shown using the most abundant seed glucosinolate 4-methylthiobutylglucosinolate (4mtb) as an example. Hydrolysis is catalyzed by β-glucosidases with thioglucosidase (myrosinase) activity. In *A. thaliana*, the above ground myrosinases TGG1 and TGG2 are known to hydrolyze glucosinolates upon tissue damage. In the presence of nitrile-specifier proteins (NSPs; left side) the unstable aglucone is converted to a nitrile, a potential substrate of nitrilases. In the absence of specifier proteins (right side), the aglucone rearranges spontaneously yielding an isothiocyanate. Isothiocyanate formation is associated with herbivore and pathogen defense while the NSP-mediated pathway has been proposed as a possible route of safe glucosinolate turnover in intact tissue. Oxidation of 4mtb catalyzed by FMO(GS-OX1) yields 4-methylsulfinylbutylglucosinolate (4msob), the most abundant leaf glucosinolate.

Glucosinolates (**Figure 1**) are characteristic specialized metabolites of the Brassicales with a well-established role in plant defense against both herbivores and pathogens (e.g. Schlaeppi et al., 2008; Bednarek et al., 2009; Clay et al., 2009; Müller et al., 2010; Stotz et al., 2011; Nakazaki et al., 2019). Due to their unusual structure that includes a sulfate group and a thioglucosidic linkage and may comprise an additional sulfur in the variable side chain (**Figure 1**), glucosinolates have long been proposed to serve as sulfur storage compounds in addition to their roles as chemical defenses (reviewed in Falk et al., 2007; Sugiyama and Hirai, 2019). In several different species, glucosinolate content has been reported to rise with sulfur supply and to decrease under sulfur deficiency (reviewed in Hirai et al., 2004; Falk et al., 2007). Moreover, glucosinolate content may decline in certain developmental stages or in response to prolonged darkness and varies diurnally (Brown et al., 2003; Falk et al., 2007; Huseby et al., 2013; Brandt et al., 2018). In support of a role in sulfur supply, seedlings of *Arabidopsis thaliana gtr1 gtr2* are smaller than wildtype seedlings when grown on medium lacking a sulfur source (Nour-Eldin et al., 2012). This mutant is deficient in the glucosinolate transporters which are, among others, responsible for transferring glucosinolates from maternal tissue to the embryo, and the seeds are devoid of glucosinolates, a potential sulfur source (Nour-Eldin et al., 2012).

Glucosinolate turnover during seed germination and seedling development has also been observed under nutrient-sufficient conditions. Inspired by the markedly different glucosinolate profiles of *A. thaliana* Col-0 seeds and plants in the rosette stage, two studies described changes of glucosinolate content and profile throughout the life cycle of *A. thaliana* Col-0 (Petersen et al., 2002; Brown et al., 2003). This showed that seeds have the highest total glucosinolate concentration among all organs of the plant (Petersen et al., 2002; Brown et al., 2003). Upon seedling development, total glucosinolate concentration decreases dramatically followed by a slight increase upon emergence and growth of the rosette leaves (Petersen et al., 2002; Brown et al., 2003; Barth and Jander, 2006). The initial decline in glucosinolate concentration could be the result of biomass gain (‘dilution’), but could also indicate a surplus of glucosinolate turnover relative to biosynthesis. In order to distinguish between these possibilities, Brown et al. (2003) determined glucosinolate content per individual during seed-seedling transition and found a net gain of glucosinolate content per individual in the first two to four days of development followed by a net decrease during the next four days (Brown et al., 2003). Together with the observed changes in glucosinolate profiles, this indicated simultaneous functioning of biosynthesis and turnover reactions with an intial surplus of biosynthesis followed by a period in which turnover outweighs total biosynthesis (Brown et al., 2003).

The defensive role of glucosinolates requires, in most cases, activation through hydrolysis by co-occuring enzymes named myrosinases (**Figure 1**). To prevent premature hydrolysis, enzyme and substrate are usually stored in separate compartments and get mixed to initiate hydrolysis. This happens either upon tissue damage (classical “mustard oil bomb”) or in intact tissue by mechanisms that are presently poorly understood (reviewed in Wittstock et al., 2016a). Classical myrosinases can be distinguished from O-glycosidases by the substitution of a catalytic Glu residue in the active site by Gln and are therefore also termed “QE-type” myrosinases (Burmeister et al., 1997). *A. thaliana* possesses six genes (*TGG1-TGG6*) encoding such myrosinases of which two are expressed in the above-ground parts (*TGG1*, *TGG2*) and two in the below-ground parts (*TGG4*, *TGG5*) of the Columbia-0 (Col-0) accession (Wittstock and Burow, 2010). Additionally, the genome of *A. thaliana* harbours a sister clade to *TGG1-TGG6* composed of sixteen genes encoding β-glucosidases (BGLU18-BGLU33), three of which have recently been demonstrated to be functional myrosinases (Bednarek et al., 2009; Clay et al., 2009; Nakano et al., 2017; Nakazaki et al., 2019). These proteins lack the amino acid substitution in the active site and are therefore also refered to as “atypical” or “EE-type” myrosinases (Nakano et al., 2017). Most members of the BGLU18–BGLU33 clade have not been characterized with respect to their activity and specificity with diverse glucosinolates as substrates. In addition, biological roles of BGLU18–BGLU33 that might be associated with activity on glucosinolates are largely unexplored with few exceptions including a role of BGLU18 in herbivore defense and a role of BGLU26 in plant innate immunity (Bednarek et al., 2009; Clay et al., 2009; Nakazaki et al., 2019; Sugiyama and Hirai, 2019).

Pathways of glucosinolate turnover have not been established, and it is unclear how stored glucosinolates would get in contact with breakdown enzymes during turnover in intact tissue. It is generally believed that turnover is initiated by hydrolysis catalyzed by myrosinases/thioglucosidases, but the responsible enzymes have not been identified. Although above-ground parts of the *tgg1 tgg2* mutant had increased concentrations of aliphatic and indolic glucosinolates two weeks after planting, a direct involvement of TGG1 and TGG2 in turnover was considered unlikely (Barth and Jander, 2006). Formation of isothiocyanates upon hydrolysis would potentially be unfavorable for the plant (Urbancsok et al., 2017). A potentially “safe” route of turnover without formation of toxic isothiocyanates would involve nitrile-specifier proteins (NSPs; Burow et al., 2009; Kissen and Bones, 2009) and nitrilases (Janowitz et al., 2009) (**Figure 1**). This would allow complete turnover of glucosinolates to building blocks for primary metabolism (glucose, sulfate, sulfur, ammonia, carboxylic acid; **Figure 1**). *A. thaliana* Col-0 expresses five *NSP* genes in different organs and developmental stages (Kissen and Bones, 2009; Wittstock et al., 2016b). Characterization of T-DNA insertion mutants of each of these genes demonstrated that simple nitrile formation depends entirely on *NSP2* in seed homogenates and mostly on *NSP1* in seedling homogenates (Wittstock et al., 2016b).

In this study, we tested the effects of deficiency in above-ground myrosinases (TGG1 and TGG2) and seed- or seedling-expressed NSPs (NSP1, NSP2) on glucosinolate levels in germinating seeds and developing seedlings in order to evaluate their possible contribution to glucosinolate turnover during seed-seedling transition. We made use of mutant lines that had been characterized previously (Barth and Jander, 2006; Wittstock et al., 2016b) and analyzed their glucosinolate content at different time points during stratification and germination in comparison to Col-0. Following the considerations by Brown et al. (2003), we determined glucosinolate content per individual (as opposed to concentrations relative to dry weight). This enabled us to identify periods in which turnover dominates (relative to *de-novo* biosynthesis) and to rule out reallocation within the plant as a possible cause of changed glucosinolate levels in a particular organ.

## Material and Methods

### Plant Material

*A. thaliana* Columbia-0, *nsp1-1* (Burow et al., 2009), *nsp2-1* and *nsp2-2* (Burow et al., 2009; Wittstock et al., 2016b) and *tgg1 tgg2* (Barth and Jander, 2006) were grown in parallel in controlled environment chambers (Percival) at 22°C, 60–70% relative humidity; 300 µmol m^−2^ s^−1^ photosynthetically active radiation (photoperiod 16 h) in several rounds between Spring 2018 and Spring 2019. After harvest, seeds were stored for six to twelve months before analysis. Each mutant-wildtype comparison was done between seed batches harvested and stored in parallel. For glucosinolate analysis, a known number of seeds (100–200) was frozen in liquid nitrogen and freeze-dried. Glucosinolate content was determined and expressed as content [nmol] per individual.

### Stratification Time Course

A known number of seeds (100–200) was incubated with 500 µl autoclaved tap water at 4°C in the dark with three replicates (randomly assigned as 1–3) for each time point. After 2, 4, 8, or 24, water was removed, samples were frozen in liquid nitrogen and freeze-dried. Glucosinolate content was determined and expressed as content (nmol) per individual. To quantify changes of glucosinolate content during imbibition, the ratio of glucosinolate content at two time points of interest was calculated for paired replicates (e.g. 8 h-replicate-1 vs. 2 h-replicate-1).

### Germination Time Course

A known number of seeds (100–200) was surface sterilized by incubation with 70% (v/v) ethanol for 2 min and 3% (v/v) sodium hypochlorite for 5 min followed by three washes with sterile water. The duration of this procedure was kept between 45 and 60 min for all samples. Seeds were plated on Murashige & Skoog (MS) medium (Murashige and Skoog, 1962) (0.9% (w/v) agar) and stratified at 4°C in the dark for 48 h. The plates were then kept in a controlled environment chamber (Percival; 22°C, 60–70% relative humidity; 300 µmol m^−2^ s^−1^ photosynthetically active radiation, photoperiod of 16 h). Plant material was harvested 4, 8, or 10 days after plating. In each of three independent experiments, three plates were generated as biological replicates (randomly assigned as 1–3) per genotype and time point. The seedlings of one plate were carefully harvested with tweezers, pooled, frozen in liquid nitrogen, and freeze-dried. Day 0 samples were frozen after sterilization without plating on MS medium. Glucosinolate content was determined and expressed as content [nmol] per individual. To quantify changes of glucosinolate content during germination, the ratio of glucosinolate content at two time points of interest was calculated for paired replicates (e.g. 10 day-replicate-1 vs. 4 day-replicate-1). Dry weight was recorded in two of the three experiments.

### Glucosinolate Analysis

Glucosinolates were quantified by HPLC of the corresponding desulfoglucosinolates (Thies, 1979) as described previously (Wittstock et al., 2016b). Desulfoglucosinolates were identified based on comparison of retention times and UV absorption spectra with those of known standards (Reichelt et al., 2002) and quantified based on peak areas at 229 nm relative to the peak area of the internal standard (4-hydroxybenzylglucosinolate; relative response factor 2.0 for aliphatic glucosinolates and 0.5 for indole glucosinolates (Burow et al., 2006; Sarosh et al., 2010). Total glucosinolate content was calculated as the sum of all detected glucosinolates (4-methylthiobutyl-(4mtb), 4-methylsulfinylbutyl-(4msob), 5-methylthiopentyl-(5mtp), 7-methylthioheptyl-(7mth), 7-methylsulfinylheptyl-(7msoh),8-methylthiooctyl-(8mto), 8-methylsulfinyloctyl-(8msoo), 3-hydroxypropyl-(3OHp), 3-benzoyloxypropyl-(3bzo), 4-benzoyloxybutyl-(4bzo), indol-3-ylmethylglucosinolate (i3m), **Table 1**). Two glucosinolates (5mtp, 3OHp) were present in traces and/or inconsistently and were therefore excluded from more detailed analyses of contents of individual glucosinolates and corresponding figures. Plant material did not contain detectable levels of 4-hydroxybutyl glucosinolate.

**Table 1:** Glucosinolates detected in seeds and germinating seedlings of *A. thaliana* and their abbreviations.

Abbreviation	Glucosinolate
4mtb	4-methylthiobutylglucosinolate
4msob	4-methylsulfinylbutylglucosinolate
5mtp	5-methylthiopentylglucosinolate
7mth	7-methylthioheptylglucosinolate
7msoh	7-methylsulfinylheptylglucosinolate
8mto	8-methylthiooctylglucosinolate
8msoo	8-methylsulfinyloctylglucosinolate
3OHp	3-hydroxypropylglucosinolate
3bzop	3-benzoyloxypropylglucosinolate
4bzob	4-benzoyloxybutylglucosinolate
i3m	indol-3-ylmethylglucosinolate

### Statistics

Statistical analyses were done with OriginPro8. Normal distribution was assumed based on Shapiro–Wilk Test. Homogeneity of variance was assessed using ANOVA with Brown–Forsythe Test. Significant differences in pairwise comparisons between wildtype and each mutant were identified using Two-Sample-t-Test (in case of normal distribution) and Mann–Whitney Test (when normal distribution was not confirmed). Multiple comparisons were done with ANOVA followed by Tukey’s test when normal distribution had been confirmed.

## Results

**Figure 2:**
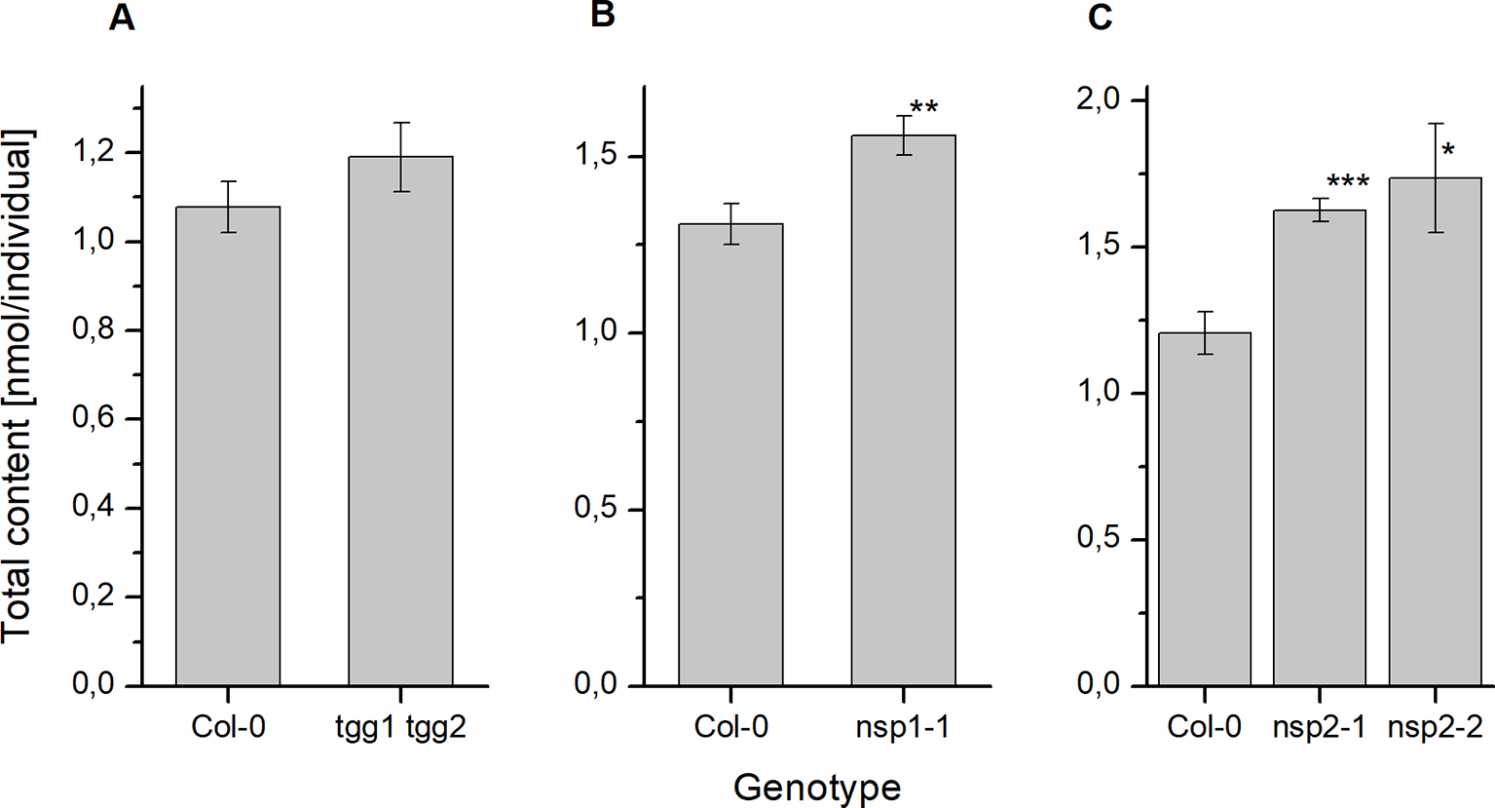
Glucosinolate content in dry seeds of *A. thaliana* Col-0 and breakdown pathway mutants. Total glucosinolate content was determined as nmol per individual in seeds of *tgg1 tgg2* **(A)**, *nsp1-1* **(B)**, as well as *nsp2-1* and *nsp2-2* **(C)** in comparison to seeds of Col-0. Means ± SD of N = 3 biological replicates. Significant differences between mutant and Col-0 are indicated by asterisks (*, p < 0.05; **, p < 0.01; Two-Sample-t-Test; ***, p < 0.001).

To investigate if deficiency in above-ground myrosinases affects glucosinolate content in seeds, we determined glucosinolate levels in *tgg1 tgg2* seeds in comparison to Col-0 wildtype. Although *tgg1 tgg2* seeds had, on average, slightly higher total glucosinolate levels per individual than Col-0 and tended to weight more than Col-0 seeds, these difference were not significant (**Figure 2**, **Figure S1**). With the exception of 4bzo, the four major seed glucosinolates (4mtb, 4bzo, 3bzo, and 8msoo; 75% of the total glucosinolate content) all contributed to the overall increase in glucosinolate content in *tgg1 tgg2* relative to Col-0 (**Figure S2**; compound abbreviations are listed in **Table 1**). Minor glucosinolates (4msob, 7mth, 7msoh, 8mto, i3m) were slightly, but not significantly increased in *tgg1 tgg2* relative to Col-0 (**Figure S2**). As glucosinolates with methylthioalkyl side chain (4mtb, 7mth, 8mto) are biosynthetic precursors of those with methylsulfinylalkyl side chain (4msob, 7msoh, 8msoo) (**Figure 1**), we also considered the total content of each of these pairs and found the level of 4mtb + 4msob to be significantly increased in *tgg1 tgg2* relative to Col-0 (**Figure S2**).

Next we studied the effect of non-functional genes of seed/seedling-expressed NSPs by assessing glucosinolate content in seeds of *nsp1-1* and two independent *nsp2* lines (*nsp2-1*, *nsp2-2*). Seeds of *nsp1-1* and both *nsp2* lines accumulated significantly higher glucosinolate levels per individual than Col-0 seeds (**Figure 2**), but their weight did not differ significantly from that of Col-0 seeds (**Figure S1**). In *nsp1-1* seeds, all glucosinolates except of 4mtb (a major glucosinolate) and 4msob were increased, on average, relative to Col-0 (**Figure S2**). The increase was significant for 7msoh 3bzo, 4bzo, and i3m (**Figure S2**). In case of *nsp2-1*, each of the methylthioalkyl/methylsulfinylalkyl pairs (4mtb + 4msob, 7mth + 7msoh, 8mto + 8msoo) as well as 4bzo, 3bzo, and i3m contributed to the overall increase (**Figure S2**). However, in case of 7mth + 7msoh and 8mto + 8msoo the increase was entirely due to a higher level of the oxidized form (methylsulfinyl-) relative to Col-0 (**Figure S2**). Seeds of *nsp2-2* showed largely the same trend as *nsp2-1*, with the exception of 8mto + 8msoo which accumulated to a lower mean total level as in Col-0 seeds (**Figure S2**).

**Figure 3:**
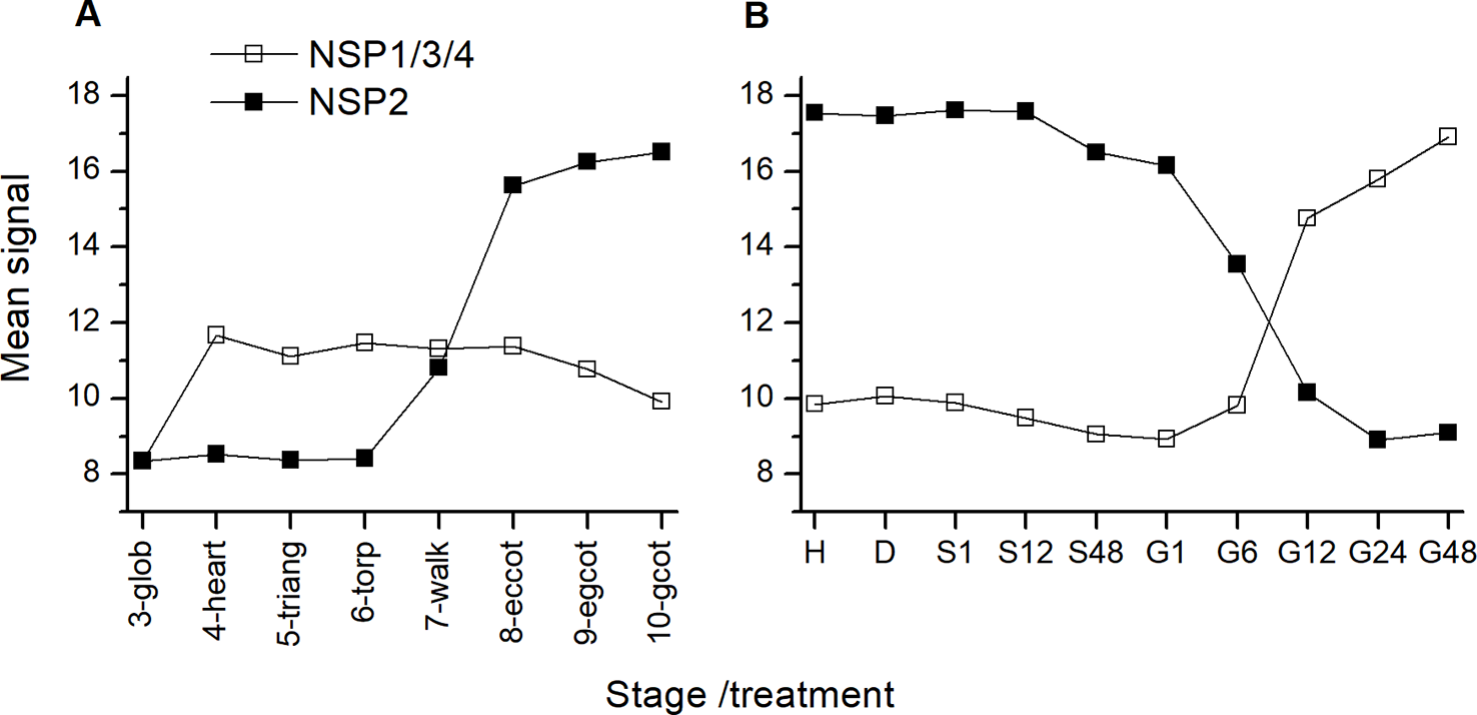
Expression of *NSP* genes during maturation and germination of *A. thaliana* Col-0 seeds. Microarray data (ATH1) from **(A)** (Kleindt et al., 2010) (GEO accession: GSE5634) and **(B)** (Narsai et al., 2011) (GEO accession: GSE30223) for *NSP1/3/4* and *NSP2* were extracted from Genevestigator (Hruz et al., 2008). Each data point represents the mean of three replicates. **(A)**: Seed maturation, samples consisted of siliques with seeds (stages 3–5) or seeds (stages 6–10). Stages are defined by embryo development according to (Kleindt et al., 2010): glob, globular to early heart; heart, early to late heart; triang, triangular (late heart to mid torpedo); torp, mid to late torpedo; walk, late torpedo to early walking stick; eccot, walking stick to early curled cotyledons; egcot, curled cotyledons to early green cotyledons; gcot, green cotyledons. **(B)**: Germination. Stages/treatments are as follows (with numbers indicating the duration of the treatment in hours): H, freshly harvested; D, dried (15 days in darkness); S, stratification (on MS plates, 4°C in the dark); G, germination (22°C, continuous light). *NSP1*, *NSP3* and *NSP4* are represented by the same probe (259381_s_at).

To investigate the effect of glucosinolate breakdown pathway genes on glucosinolate levels during germination, we conducted two different treatments. Treatment 1 addressed early timepoints within up to 72 h of stratification and germination. Therefore, the procedure was kept simple. We subjected seeds to imbibition in water at 4°C in the dark and measured glucosinolate content per individual after 2, 6, 8, 12, 24, 48, and (in some experiments) 72 h. We conducted different sets of experiments with either *tgg1 tgg2* and Col-0 or both *nsp2* lines and Col-0. The *nsp2* lines were chosen as *NSP2* is expressed in seeds and during stratification (**Figure 3**), and as simple nitrile formation in seed homogenates depends entirely on *NSP2* (Wittstock et al., 2016b). Treatment 2 considered changes in glucosinolate content during the standard procedure for germination of *A. thaliana* seeds on MS medium under sterile conditions over a total of ten days and included sterilization, plating, stratification for two days and incubation at 22°C and with 16 h photoperiod for another eight days. In these experiments, we analyzed *tgg1 tgg2* and *nsp1-1* in comparison to Col-0. The *nsp1-1* line was chosen as the transfer from stratification to normal growth conditions is associated with a switch from *NSP2* to *NSP1/3/4* expression (**Figure 3**) and *NSP1* is expressed in seedlings (in contrast to *NSP3* and *NSP4*) (Wittstock et al., 2016b).

**Figure 4:**
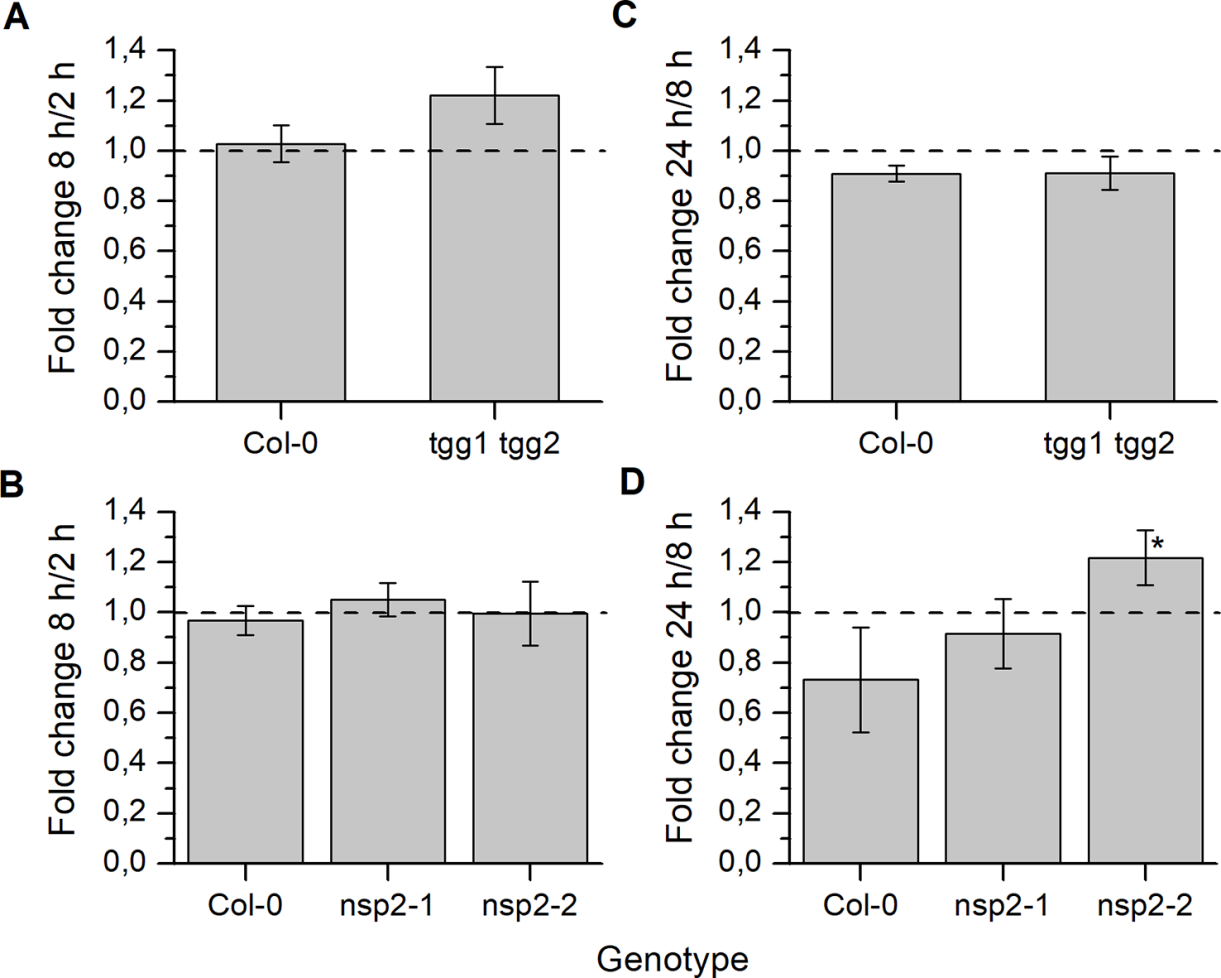
Changes of seed glucosinolate content during stratification. Seeds of Col-0 and *tgg1 tgg2* **(A**, **C)** or Col-0, *nsp2-1*, and *nsp2-2* **(B**, **D)** were incubated in autoclaved tap water at 4°C in the dark for 2, 8, or 24 h. Total glucosinolate content was determined as nmol per individual. Fold change from 2 h to 8 h **(A**, **B)** and from 8 h to 24 h **(C**, **D)** is given. Ratio of 1 (no change) is highlighted by the dashed line. Means ± SD of N = 3 biological replicates. Significant differences between mutant and Col-0 are indicated by asterisks (*, p < 0.05, Two-Sample-t-Test).

Based on the overall time course in Treatment 1 (**Figure S3**), we selected three time points (2, 8 and 24 h) for comparison between Col-0 and mutants. To quantify changes, we calculated the ratio (fold change) between the content at a later and an earlier time point (**Figure 4**). Thus, values >1 indicate an increase of glucosinolate content over time, and values <1 a decrease. In *tgg1 tgg2* seeds, glucosinolate content after 8 h was, on average, about 1.2-fold higher than after 2 h of incubation (**Figure 4**), but this difference was not significant (p > 0.05, Mann–Whitney Test). Glucosinolate content in seeds of Col-0 and both *nsp2* lines did not change significantly from 2 to 8 h of incubation (**Figure 4**). The 8h/2h ratio did not differ significantly between Col-0 and the mutant lines (**Figure 4**). Between 8 and 24 h of incubation, glucosinolate content of *nsp2-2* seeds increased about 1.2-fold (p < 0.05, Two-Sample-t-Test) while the content in seeds of the other tested genotypes decreased on average (**Figure 4**). The 24 h/8 h ratio differed significantly between *nsp2-2* and Col-0 (p < 0.05, Two-Sample-t-Test) (**Figure 4**).

**Figure 5:**
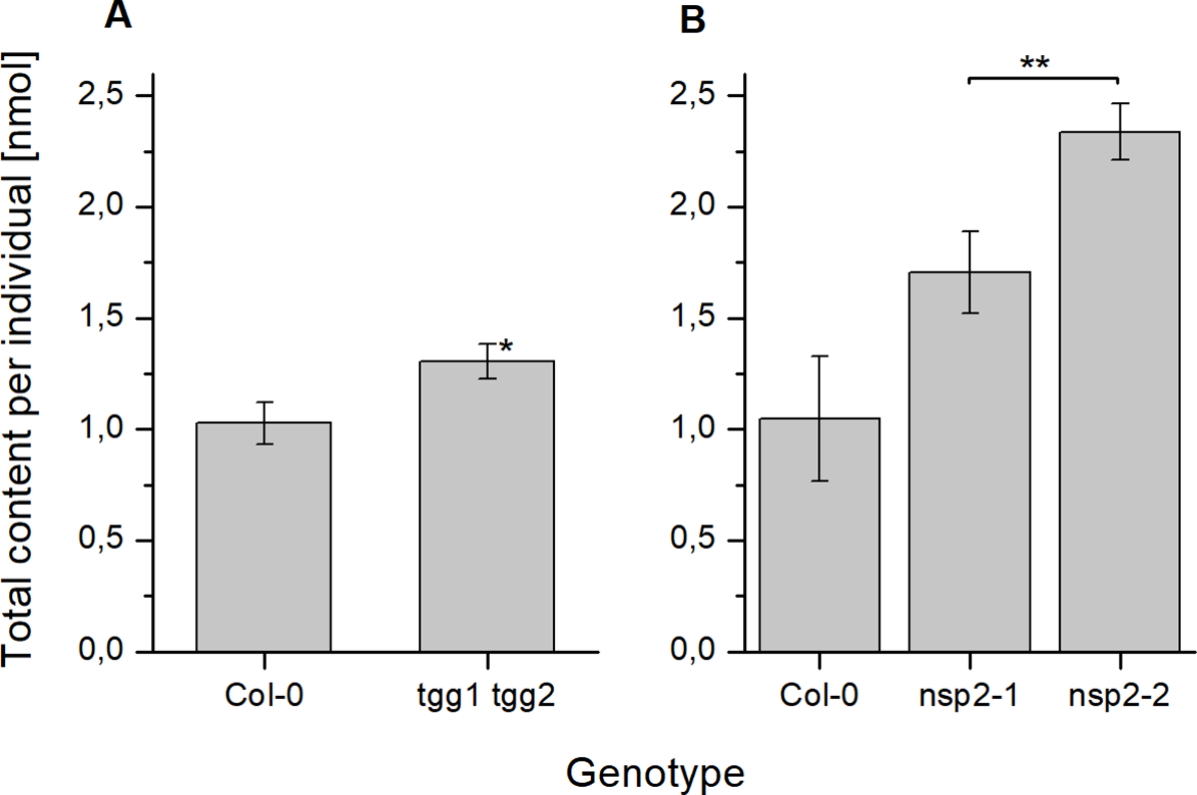
Seed glucosinolate content after 24 h of stratification. Seeds were incubated in autoclaved tap water at 4°C in the dark for 24 h. Total glucosinolate content was determined as nmol per individual in seeds of *tgg1 tgg2* **(A)** and *nsp2-1* and *nsp2-2* **(B)** in comparison to seeds of Col-0. Means ± SD of N = 3 biological replicates. An asterisk next to a bar indicates a significant difference to Col-0. Asterisks at a horizontal bracket denote a significant difference between the bars. *, p < 0.05; **, p < 0.01 (Two-Sample-t-Test).

**Figure 6:**
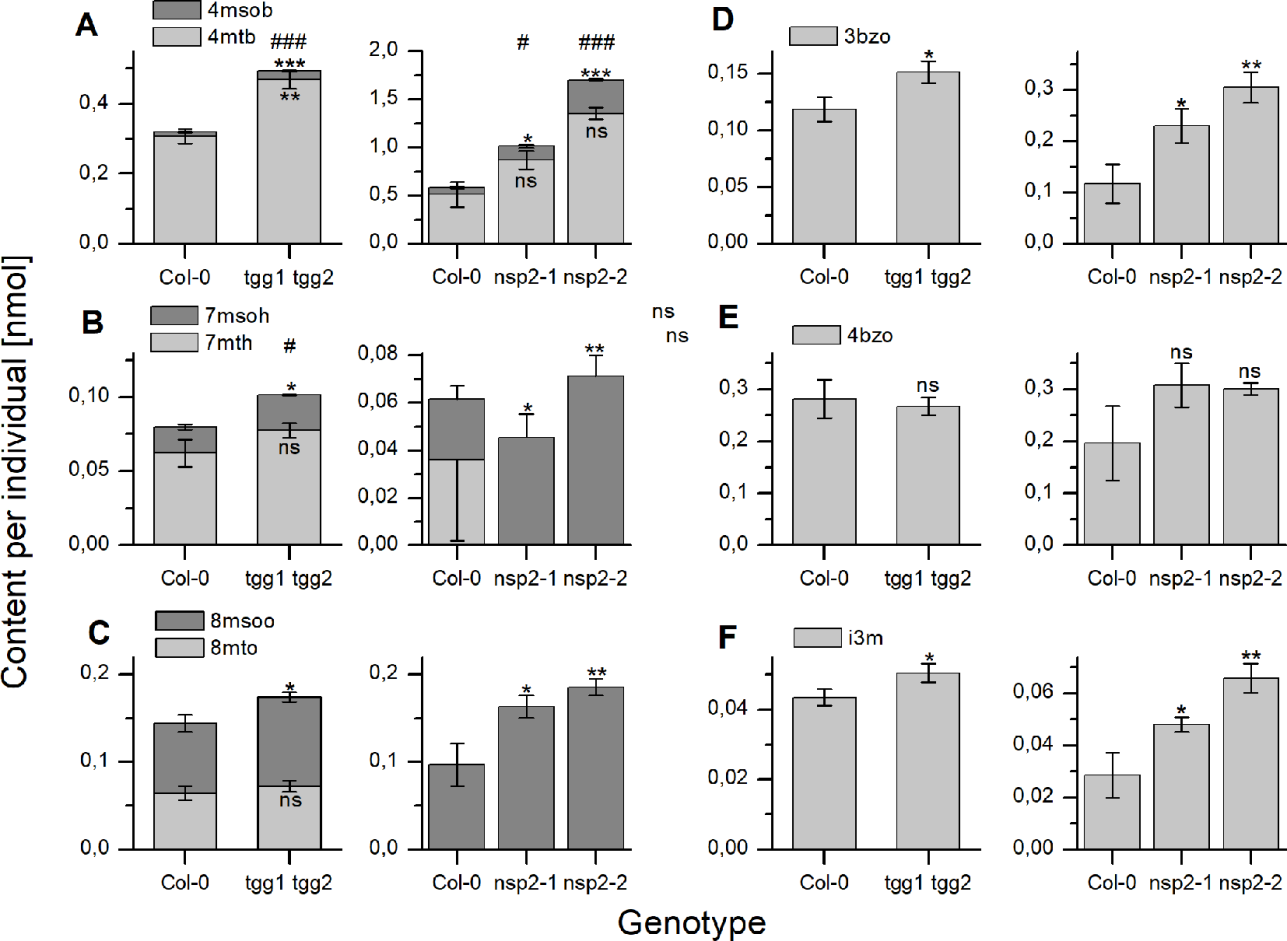
Seed content of individual glucosinolates after 24 h of stratification. Seeds were incubated in autoclaved tap water at 4°C in the dark for 24 h. Glucosinolate content is expressed as nmol per individual. **(A**–**C)** depict methylthioalkylglucosinolates together with the derived methylsulfinylalkyl derivative. **(D**, **E)** depict individual benzoylated glucosinolates and **(F)** indol-3-ylmethylglucosinolate. The left graph of each panel shows results of experiments with *tgg1 tgg2* in comparison with Col-0, the right graph in each panel those with two independent *nsp2* lines in comparison with Col-0. Means ± SD from N = 3 biological replicates. Asterisks indicate significant differences for individual glucosinolates relative to Col-0 (*, p < 0.05; **, p < 0.01; ***, p < 0.001) while hashs on top of a bar in **(A**–**C)** indicate significant differences for the sum of a biosynthetically linked pair relative to wildtype (#, p < 0.05; ###, p < 0.001). Significant differences were identified by pairwise comparisons using Two-Sample-t-Test with the exception of 4mtb in Col-0 vs. both *nsp2* mutants (A, right graph). In the latter case, Mann–Whitney Test was applied. Levels of 7mth **(B)** and 8mto **(C)** were low, varied strongly and could not be detected in some samples of Col-0 as well as *nsp2-1* and *nsp2-2*. ns, not significant.

As a consequence of these changes, *tgg1 tgg2* seeds contained significantly higher total glucosinolate levels per individual than Col-0 after 24 h of stratification (**Figure 5**). On average, individual glucosinolates were all increased in *tgg1 tgg2* relative to Col-0 with the exception of 4bzo (**Figure 6**), similar to the results with dry seeds (**Figure S2**). Seeds of *nsp2-2* had the highest glucosinolate content among the tested genotypes at this time point and contained, on average, >2.3-fold higher levels per individual than Col-0 seeds after the same treatment (**Figure 6**). All individual glucosinolates were increased, on average, in *nsp2-2* seeds compared to Col-0 (**Figure 6**) with the exception of 7mth which was replaced by 7msoh in *nsp2-2* seeds, i.e. the level of 7msoh in *nsp2-2* was higher than the level of 7mth + 7msoh in Col-0 (**Figure 6B**). The proportion of reduced and oxidized form (7mth/7msoh, 8mto/8msoo) also varied in Col-0 seeds (**Figures 6B, C**). These four glucosinolates were of low abundance. Together with differences between seed batches, this might explain the large variation. Seeds of *nsp2-1* showed the same trend as those of *nsp2-2* with respect to total glucosinolate content (**Figure 5**) and individual glucosinolates (**Figure 6**). Taken together, the differences of total glucosinolate content in seeds between Col-0, *tgg1 tgg2* and both *nsp2* lines determined with dry seeds became more pronounced when seeds were stratified in water at 4°C in the dark for 24 h.

**Figure 7:**
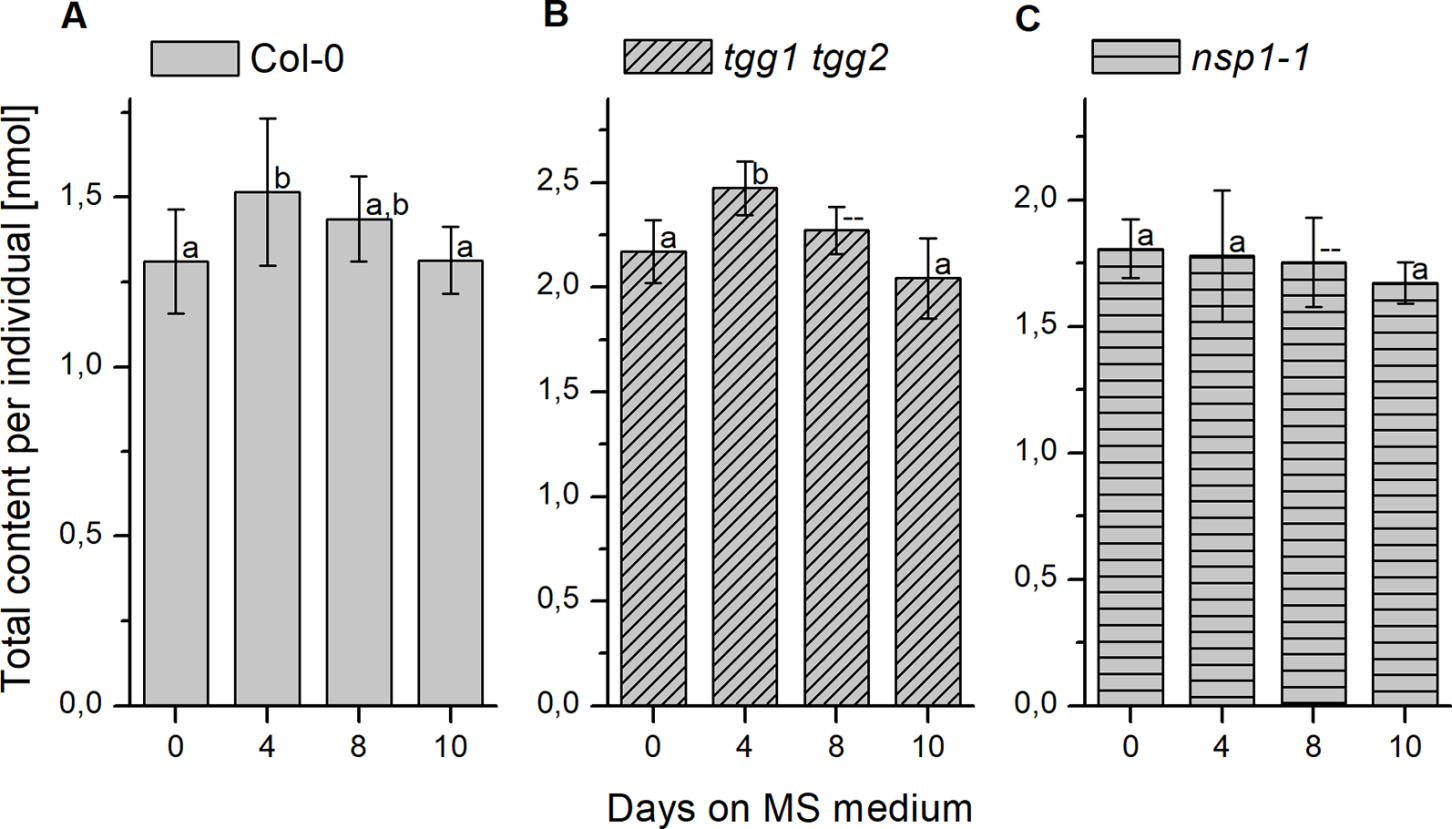
Changes of glucosinolate content in sterilized seeds plated on MS medium. Seeds were sterilized (day 0), plated on MS medium, stratified at 4°C in the dark for two days and then grown at 22°C with 16 h photoperiod. Total glucosinolate content was determined as nmol per individual in seeds of Col-0 **(A)**, *tgg1 tgg2* **(B)**, as well as *nsp1-1* **(C)**. Means ± SD (N = 8 – 9). Different letters above bars indicate a significant difference (p < 0.05, ANOVA with Tukey’s test; data for day 8 in **(B)** and **(C)** were not included as normal distribution was not confirmed).

**Figure 8:**
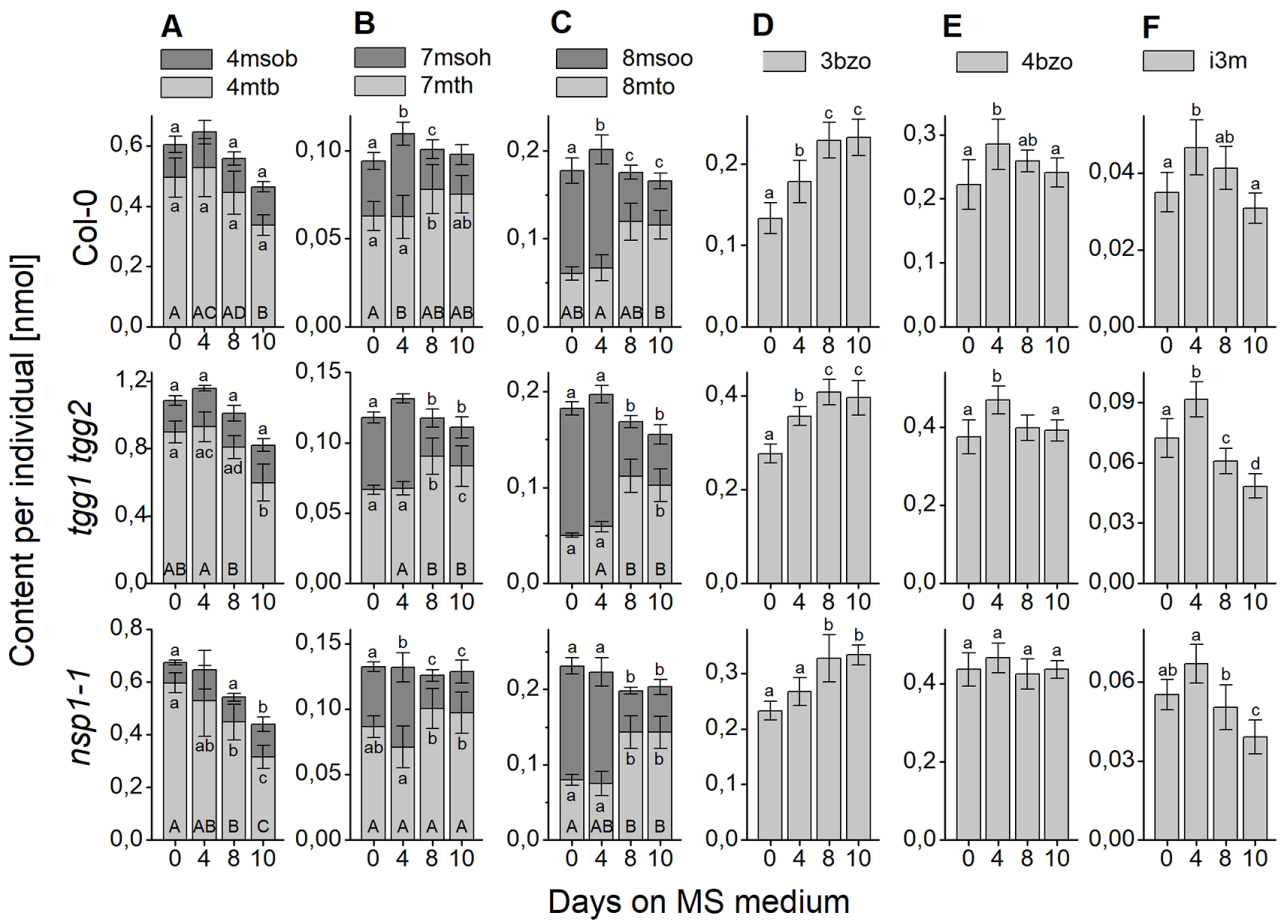
Changes of content of individual glucosinolates in sterilized *A. thaliana* Col-0 seeds plated on MS medium. Seeds were sterilized (day 0), plated on MS medium, stratified at 4°C in the dark for two days and then grown at 22°C with 16 h photoperiod. Glucosinolate content was determined as nmol per individual. **(A**–**C)** depict methylthioalkylglucosinolates together with the derived methylsulfinylalkyl derivatives. **(D**, **E)** depict individual benzoylated glucosinolates and **(F)** indol-3-yl-methylglucosinolate. Each panel shows a comparison of Col-0 (top), *tgg1 tgg2* (middle), and *nsp1-1* (bottom). Means ± SD from N = 8–9 biological replicates. Different letters next to error bars indicate a significant difference for individual glucosinolates between time points while those at the base of bars indicate significant differences for the sum of biosynthetically linked pairs (p < 0.05, ANOVA with Tukey’s test; samples without a label were not included in the analysis as normal distribution could not be assumed).

Changes of seed glucosinolate content during germination (Treatment 2) were assessed at four time points after sterilization during up to ten days of growth on MS medium. For Col-0 seeds, we were able to reproduce, in principle, the previously published time course (Brown et al., 2003) with a peak in total glucosinolate content per individual at day 4 and, in our study, no significant difference between total glucosinolate content at days 0 and 10 (**Figure 7A**). The content of most individual glucosinolates or biosynthetically linked pairs (7mth + 7msoh, 8mto + 8msoo, i3m), respectively, showed the same trend (**Figure 8**, top row from left to right), similar to a previous report (Brown et al., 2003). However, the major glucosinolate 3bzo increased steadily over the investigated time period to reach an almost twofold higher level at day 10 when compared with day 0 (**Figure 8D**). The content of the major seed glucosinolate 4mtb and the sum of 4mtb and its derivative 4msob declined below the level at day 0, in our study to about 70% (4mtb) and 80% (4mtb + 4msob) of the content at day 0 indicating turnover (**Figure 8A**). In case of the other two methylthio-/sulfinylalkylglucosinolate pairs, we found increased levels of the methylthioalkyl compounds (7mth, 8mto) at days 8 and 10 relative to day 4, but decreased levels of their oxidized derivatives (7msoh, 8msoo) (**Figures 8B, C**). Thus, the oxidized forms might undergo turnover or are converted back to the methylthioalkyl precursors.

The analysis of germinating seeds of *tgg1 tgg2* resulted in a similar time course of glucosinolate content as that obtained for Col-0 seeds (**Figure 7B**). There was no significant difference between *tgg1 tgg2* and Col-0 with respect to the ratio (fold change) between days 0 and 4 as well as between days 4 and 10 (p > 0.05, Two-sample-t-Test, **Figure S4**). The time course of each individual glucosinolate (**Figure 8**, middle row from left to right) largely mirrored that found with Col-0 seeds This indicates that TGG1 and TGG2 are not involved in the reactions that determine the time course of glucosinolate content in germinating *A. thaliana* seeds within 10 days of incubation on MS medium.

In contrast to Col-0 and *tgg1 tgg2*, germinating seeds of *nsp1-1* did not contain higher total glucosinolate levels on day 4 relative to days 0 and 10 (**Figure 7C**). Fold change between days 0 and 4 was significantly different from that obtained with Col-0 (p <0.05, Two-sample-t-Test, **Figure S4**). On average, total glucosinolate content of *nsp1-1* seeds decreased slightly and steadily over 10 days of incubation on MS medium, but these changes were not significant (p > 0.05, ANOVA; **Figure 7C**). Among individual glucosinolates, time courses of 3bzo and i3m were similar to those found with Col-0 while time courses of all other glucosinolates lacked the peak at day 4 (**Figure 8**, bottom row from left to right). Our results indicate that the time course of glucosinolate content in *A. thaliana* Col-0 seeds upon 10 days of growth on MS medium depends on functional *NSP1*.

To assess if the observed differences between the genotypes can be explained by a developmental defect, we inspected the developing seedlings of Col-0, *tgg1 tgg2*, and *nsp1-1* visually and determined their dry weight over ten days of growth on MS medium (**Figures S5** and **S6**). At day 4, seedlings of all genotypes had emerged with light-green cotyledons and a primary root, and cotyledons had started to open (**Figure S5**). At day 8, seedlings possessed opened, green cotyledons (**Figure S5**). The plumule started to become visible with the naked eye. Cotyledons and plumule tended to appear slightly larger in seedlings of *tgg1 tgg2* than in the other genotypes (**Figure S5**). At day 10, *tgg1 tgg2* seedlings were larger than seedlings of the other genotypes and had typically developed the first pair of true leaves while only few seedlings of the other genotypes had reached this stage (**Figure S5**). Thus, *tgg1 tgg2* developed slightly faster than the other genotypes. In agreement with this, *tgg1 tgg2* had, on average, the highest dry weight among the tested genotypes throughout days 4 to 10 (**Figure S6**). Col-0 seedlings had the lowest dry weight and *nsp1-1* seedlings reached intermediate levels (**Figure S6**) with the exception of day 8 where dry weight of *nsp1-1* was the same as that of *tgg1 tgg2*. Taken together, we found *tgg1 tgg2* to gain weight and develop slightly faster than Col-0 and *nsp1-1*, but we did not find developmental differences among the genotypes that could explain the changed time course of glucosinolate content in *nsp1-1* relative to Col-0 and *tgg1 tgg2*.

## Discussion

While turnover of seed glucosinolates during germination and seedling development of *A. thaliana* has been described previously based on different experimental approaches (Petersen et al., 2002; Brown et al., 2003; Barth and Jander, 2006) and was confirmed here (**Figure 7**), pathways of glucosinolate turnover have remained unresolved. The present study was set up to evaluate the time course of glucosinolate content during stratification and germination in dependency of enzymes known to be involved in glucosinolate breakdown upon tissue damage, namely TGG1, TGG2, NSP1 and NSP2, using previously characterized T-DNA mutants. The *tgg1 tgg2* mutant lacked myrosinase activity in the above-ground parts of plants in the vegetative as well as generative stages when myrosinase activity of plant extracts was determined with allylglucosinolate as substrate but had residual myrosinase activity on indolic glucosinolates (Barth and Jander, 2006). TGG1 seems to be the major myrosinase of the above-ground organs because the single mutant *tgg1* gave similar results as the double mutant while results for *tgg2* were similar to wildtype (Barth and Jander, 2006). *NSP2* is expressed only in seeds and solely responsible for simple nitrile formation in Col-0 seed homogenates without contributions from other *NSPs* (Wittstock et al., 2016b). Under laboratory conditions, the switch from *NSP2* to *NSP1* expression coincides with the transfer from stratification to germination (**Figure 3**). However, *NSP2* expression seems to be confined to late stages of seed maturation and to the mature seed until the onset of germination (**Figure 3**). Among non-functional alleles of *NSP1–NSP5*, only the *nsp1-1* allele caused a significant decrease of absolute and relative levels of the simple nitrile and a significant increase of the corresponding isothiocyanate derived from 4mtb (the most abundant aliphatic glucosinolate) in seedling homogenates, but did not result in a complete lack of simple nitrile formation (Wittstock et al., 2016b). Hence, other NSPs are likely to contribute, albeit to a low extent. It would, therefore, be interesting to include mutants with multiple non-functional *NSP* alleles in future studies on glucosinolate turnover. As *NSP1*, *NSP3*, and *NSP4* are tandem genes, a triple mutant cannot be obtained by crossing of the existing genotypes but has to be generated by alternative methods, e.g. gene editing. In *nsp5-1*, *NSP5* expression was downregulated, but not lacking (Wittstock et al., 2016b). Once a real knockout has been obtained, it should also be studied in a background with other non-functional *NSPs*.

We did not find differences between *tgg1 tgg2* and Col-0 with respect to the time course of total glucosinolate content over 10 days of incubation on MS medium (**Figure 7**, **Figure S4**). Thus, TGG1/TGG2 are not responsible for the decline of glucosinolate content between days 4 and 10 of the germination time course in agreement with the conclusions drawn by Barth and Jander (2006). However, glucosinolate content in dry seeds of *tgg1 tgg2* was, on average, higher than that of Col-0, and after 24 h of stratification this difference was larger and significant (**Figures 2A** and **5A**). As a possible explanation, TGG1/TGG2 could be involved in breakdown of glucosinolates in developing siliques/seeds, for example for the release of volatile breakdown products. In fact, *TGG1/TGG2* are expressed in early stages of silique development (stages 3–6, Kleindt et al., 2010) according to microarray data (GEO: GSE5634; *TGG1* and *TGG2* are represented by the same probe). Lacking *TGG1/TGG2* expression could also have an effect on glucosinolate import into the developing seed or biosynthesis. Even though we did not observe higher herbivore or pathogen infestation rates of *tgg1 tgg2* plants as compared to simultaneously grown Col-0 plants, defense pathways could have been induced in the mutant relative to Col-0 leading to enhanced glucosinolate biosynthesis in the parent plants of the analyzed *tgg1 tgg2* seeds. Upon stratification in water, we observed a steep increase of glucosinolate content in *tgg1 tgg2* between 6 and 10 h of incubation, and the resulting difference in content relative to Col-0 remained largely unchanged for the following 40 h (**Figure S3**). This might indicate that glucosinolate breakdown by TGG1/TGG2 happens only within a small time window during stratification (maybe due to reorganization of tissues upon onset of imbibition), but is normally compensated by biosynthesis. Further experiments will need to be performed to investigate this possibility in more detail. Seedlings of *tgg1 tgg2* grew faster than Col-0 seedlings. It is tempting to speculate that this could be due to the benefit provided by increased levels of glucosinolates as substrates fed into TGG1/TGG2-independent turnover pathways for nutrient release. As an alternative explanation, lack of expression of TGG1, a highly abundant protein in wildtype plants, might safe considerable resources which *tgg1 tgg2* plants can invest in growth.

**Figure 9:**
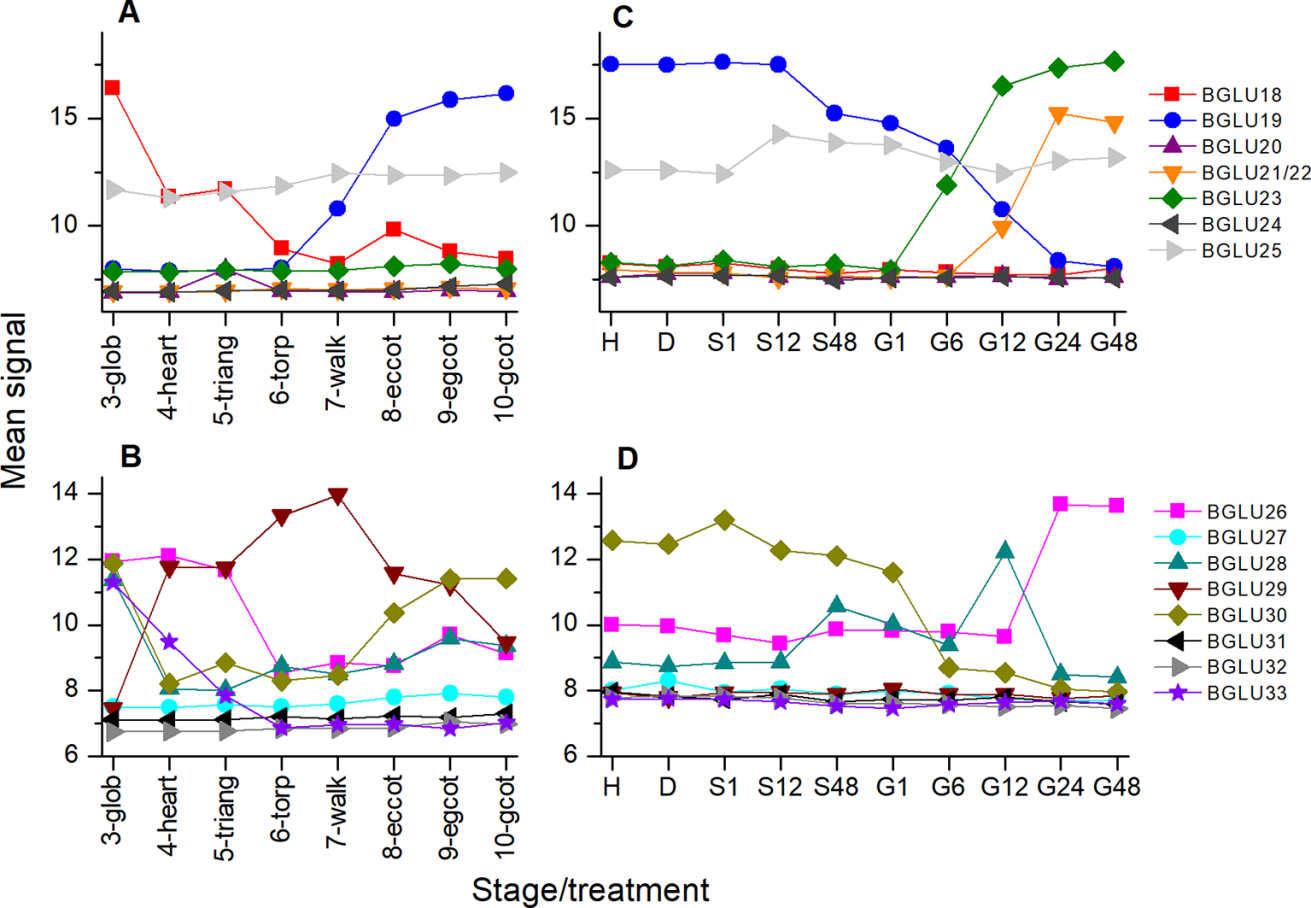
Expression of *BGLU* genes during maturation and germination of *A. thaliana* Col-0 seeds. Microarray data (ATH1) from (Kleindt et al., 2010) (GEO accession: GSE5634) **(A**, **B)** and (Narsai et al., 2011) (GEO accession: GSE30223) **(C**, **D)** for *BGLU18-25* **(A**, **C)** and *BGLU26-BGLU33* **(B**, **D)** were extracted from Genevestigator (Hruz et al., 2008). Each data point represents the mean of three replicates. **(A**, **B)** Seed maturation, samples consisted of siliques with seeds (stages 3–5) or seeds (stages 6–10). Stages are defined by embryo development according to (Kleindt et al., 2010): glob, globular to early heart; heart, early to late heart; triang, triangular (late heart to mid torpedo); torp, mid to late torpedo; walk, late torpedo to early walking stick; eccot, walking stick to early curled cotyledons; egcot, curled cotyledons to early green cotyledons; gcot, green cotyledons. **(C**, **D)** Germination. Stages/treatments are as follows (with numbers indicating the duration of the treatment in hours): H, freshly harvested; D, dried (15 days in darkness); S, stratification (on MS plates, 4°C in the dark); G, germination (22°C, continuous light). *BGLU21* and *BGLU22* are represented by the same probe (260130_s_at).

If TGG1/TGG2 are not responsible for the observed decline in glucosinolate content between days 4 and 10 of the germination time course, as our data suggest, other enzymes must initiate glucosinolate turnover. To test if any of the genes of the *BGLU18-BGLU33* cluster are expressed in germinating seeds, we analyzed microarray data using Genevestigator (Hruz et al., 2008) (**Figures 9C, D**). This identified *BGLU21/BGLU22*, *BGLU23*, *BGLU26*, and *BGLU28* to be induced upon transfer to light. All of these genes except for *BGLU28* reached the highest expression level at 24–48 h of incubation in light, i.e. days 3 to 4 of incubation on medium, and are therefore candidates for future studies on glucosinolate turnover in germinating seeds of *A. thaliana*. Based on the present knowledge, BGLU23 and BGLU26 are active on indolic glucosinolates, and BGLU23 is unable to hydrolyze allylglucosinolate (Bednarek et al., 2009; Clay et al., 2009; Ahn et al., 2010; Nakano et al., 2017). However, a broad spectrum of structurally diverse glucosinolates needs to be tested to evaluate possible roles of these and other atypical myrosinases in processes such as glucosinolate turnover during seedling development.

As NSPs act downstream of glucosinolate hydrolysis (**Figure 1**; (Eisenschmidt-Bönn et al., 2019), we did not expect large effects of mutations in *NSP* genes on glucosinolate content and its time course during germination. Surprisingly, we found non-functional alleles of *NSP1* and *NSP2* to have significant effects on glucosinolate content in dry seeds, seeds stratified in water and germinating seeds, respectively. Mutant lines deficient in *NSP1* or *NSP2* had significantly higher glucosinolate contents in seeds than Col-0 (**Figures 2B, C**), but did not differ from Col-0 seeds in weight (**Figure S1**). As the two NSPs are expressed at different time points during seed development (**Figure 3**), we assume that the high glucosinolate content is due to effects exerted at different stages of seed development including effects on the mother plant. For example, NSP1 could be involved in turnover reactions that take place during early silique development, while NSP2 would act at later time points of seed maturation. If these NSPs are involved in turnover pathways by converting the glucosinolate aglucone to a nitrile (**Figure 1**), our results could indicate downregulation of glucosinolate hydrolysis in response to accumulation of undesirable breakdown products, e.g. isothiocyanates. Besides *TGG1/TGG2*, several *BGLUs* of the BGLU18–BGLU33 clade are expressed in early stages of silique development (*BGLU18*, *BGLU26*, *BGLU28*, *BGLU29*, *BGLU30*, *BGLU33*; **Figure 9**) and could be affected by *NSP1* deficiency. At the later stages of seed maturation, when *NSP2* is expressed, *BGLU19* and *BGLU30* are induced in Col-0 (**Figure 9**). Future experiments will have to show if these genes are downregulated in developing seeds of the *nsp2* mutant lines. There is presently no experimental support for a regulatory role of NSP1 and NSP2 that is independent of their enzymatic activity, but we cannot rule out alternative mechanisms of action based on current knowledge.

In our stratification time course experiments with *nsp2* mutants, we found elevated glucosinolate levels in both *nsp2* lines relative to Col-0 at 24 h of incubation in water at 4°C in the dark (**Figure 5**). This was, on one hand, a consequence of the increased content in the seeds. On the other hand, we found glucosinolates to increase in *nsp2-2* especially between 8 and 24 h of incubation while glucosinolate content decreased in Col-0. Although only one of the *nsp2* lines showed this increase, this result might again indicate that a non-functional pathway of nitrile formation may result in downregulation of hydrolysis. At 24 h of stratification, *TGG1/TGG2* as well as *BGLU19*, *BGLU28* and *BGLU30* could be involved (**Figure 9**).

The germination time course experiments with *nsp1-1* showed that *NSP1* deficiency affects the time course significantly (**Figure 7**, **Figure S4**). While total glucosinolate content increased in Col-0 seeds between days 0 and 4 of germination on MS medium and then decreased, the content in *nsp1-1* seeds did not increase or decrease over the entire observation period (**Figure 7**). If *NSP1* is involved in turnover during germination, its lacking expression appears to have a different effect at this stage when compared to seeds or lacking *NSP2* expression during stratification. But as indicated above and illustrated in **Figure 9**, other BGLUs might be involved in glucosinolate turnover during germination than during seed maturation and stratification, and maybe they react differently to the inability to form nitriles. Additionally, genes/enzymes involved in biosynthesis could be affected as well, and biosynthesis could be downregulated as a consequence of *NSP1* deficiency. Of note, accumulation of isothiocyanates (instead of nitriles) would likely reduce the amount of glutathione available for glucosinolate biosynthesis as isothiocyanates readily react with glutathione, either spontaneously or enzymatically catalyzed (Czerniawski and Bednarek, 2018; Urbancsok et al., 2018).

In addition to the evaluation of total glucosinolate contents, we have also recorded the levels of the most abundant individual glucosinolates and compared them between different time points and the different genotypes analyzed. Although we found individual glucosinolates or biosynthetically linked pairs of glucosinolates to largely mirror the trends found for total glucosinolate content in mutants vs. Col-0, we noticed some interesting exceptions. For example, *tgg1 tgg2* had increased total glucosinolate contents in dry seeds and after 24 h of stratification in water compared to Col-0, but the level of 4bzo did not differ between the two genotypes (**Figure S2**, **Figure 6**). In case of *nsp1-1*, 4mtb+4msob did not contribute to the increased glucosinolate content in dry seeds relative to Col-0, but 3bzo and 4bzo were increased overproportionally in *nsp1-1* relative to Col-0 (**Figure S2**). During germination, the levels of 3bzo and i3m in *nsp1-1* underwent a time course similar to Col-0 while all other glucosinolates and the total glucosinolate content did not change in *nsp1-1* (**Figure 8**). At present, we can only speculate that these observations might be explained by different substrate specificities of the hydrolytic enzymes involved in turnover pathways or by specific localization of certain glucosinolates in some stages of development.

Although our data confirm glucosinolate turnover during seed-seedling transition and provide some indication for involvement of NSPs, more experiments are required to establish a direct participation of NSPs in turnover pathways. For example, experiments should be directed at capturing metabolites formed specifically in developing and germinating seeds of mutants impaired in nitrile formation, but not or to lesser extent in Col-0. Furthermore, the effects of non-functional *NSP* alleles on expression of other genes involved in glucosinolate breakdown and glucosinolate biosynthesis should be studied in detail in these stages. In order to establish pathways of glucosinolate turnover, it would also be essential to biochemically characterize more BGLUs of the BGLU18–BGLU33 cluster to find out if they accept glucosinolates as substrates. It has to be clarified how substrate and enzyme(s) come together in intact tissue to allow hydrolysis of glucosinolates to occur with a high level of spacial and temporal control. Although the analysis of whole individuals allowed us to rule out reallocation between organs as a possible course of changed glucosinolate content, we might have missed tissue-specific effects (e.g. a surplus of turnover in one tissue balanced out by a surplus of biosynthesis in another tissue) in this setup. The design of experiments directed at tissue specific variation will be facilitated by a better knowledge of genes and enzymes involved in turnover pathways. Last but not least, the physiological roles of glucosinolate turnover in germinating seeds deserves more attention. Apart from a role in nutrient supply [which has been discussed controversially (Aghajanzadeh et al., 2014)], turnover could represent a mechanism of regulating glucosinolate levels or it could generate signals upon which biosynthesis rates are adjusted. Interestingly, glucosinolate concentrations per unit dry weight differed significantly between Col-0, *tgg1 tgg2* and *nsp1-1* at day 0 of the germination time course, but were equal at day 10 (**Figure S7**). This would be in agreement with mechanisms that allow the plant to sense glucosinolate concentration and to maintain it at a fixed level as suggested previously (Brown et al., 2003).

The presence and abundance of different glucosinolate types and the structural outcome of glucosinolate hydrolysis in tissue homogenates are subject to an enormous natural variation (Lambrix et al., 2001; Witzel et al., 2015; Wentzell and Kliebenstein, 2008). It would, therefore, be worthwhile to extend investigations on glucosinolate turnover during seed-seedling transition to other accessions of *A. thaliana* and to other species of the Brassicaceae as well as other families of the Brassicales. To our knowledge, natural variation of the *NSP* genes in *A. thaliana* or other Brassicaceae has not been assessed systematically yet. In case of the epithiospecifier protein (ESP), a large proportion of the *A. thaliana* accessions analyzed so far (including Col-0) does not possess a functional allele at the *ESP* locus. The presence of a functional allele seems to correlate with the presence of alkenyl glucosinolates (Lambrix et al., 2001) which give rise to formation of epithionitriles upon hydrolysis in the presence of ESP. It remains to be tested if both NSPs and ESP affect glucosinolate turnover in *A. thaliana* accessions with alkenyl glucosinolates as predominant glucosinolates. Interestingly, there is presently no indication for the presence of functional specifier proteins in other families than the Brassicaceae (Kuchernig et al., 2012). This raises the question if turnover of seed glucosinolates during germination is a general phenomenon of glucosinolate-producing plants and if so, if the mechanisms are evolutionarily conserved. In the absence of specifier proteins, isothiocyanates are likely to be formed as initial hydrolysis products upon turnover. The ability to generate nitriles instead and to recycle them into primary metabolism could potentially represent an innovation that contributed to the evolutionary success of the Brassicaceae.

In conclusion, our study demonstrates that the decline of glucosinolate content in *A. thaliana* Col-0 seeds during germination (days 4 to 10) does not depend on functional TGG1 and TGG2. This indicates involvement of other enzymes, e.g. members of the BGLU18–BGLU33 clade of β-glucosidases, in glucosinolate turnover at this stage of development. Furthermore, the observed time course with highest glucosinolate content at day 4 of germination requires functional *NSP1*. Increased glucosinolate levels in dormant and stratified seeds deficient in TGG1/TGG2, NSP1 or NSP2 suggest turnover to take place during seed maturation and stratification and to depend on these enzymes. However, more research is required to provide evidence for a direct participation of TGG1/TGG2, NSP1 and NSP2 in corresponding turnover pathways. Based on our study, we hypothesize that different enzymes are involved in such pathways at different stages of seed maturation and germination.

## Data Availability Statement

All datasets generated for this study are included in the article/**Supplementary Material**.

## Author Contributions

UW conceived the study. KM and ME designed and conducted experiments. KM, ME, and UW analyzed data. UW and ME performed statistical analysis. UW analyzed gene expression data. UW wrote the manuscript. All authors contributed to manuscript revision, read and approved the submitted version.

## Funding

This research was funded by Technische Universität Braunschweig. We acknowledge support by the German Research Foundation and the Open Access Publication Funds of the Technische Universität Braunschweig.

## Conflict of Interest

The authors declare that the research was conducted in the absence of any commercial or financial relationships that could be construed as a potential conflict of interest.
