# Supplementary material for: Glucosinolate Content in Dormant and Germinating Arabidopsis thaliana Seeds Is Affected by Non-Functional Alleles of Classical Myrosinase and Nitrile-Specifier Protein Genes
Source: Front Plant Sci. 2019 Nov 26;10:1549. doi: 10.3389/fpls.2019.01549 (PMC6901928; doi:10.3389/fpls.2019.01549)
Supplement: Supplementary file 1 [file Presentation_1.pdf]

Supplementary Material

Glucosinolate content in dormant and germinating *Arabidopsis thaliana* seeds is affected by non-functional alleles of classical myrosinase and nitrile-specifier protein genes

Running title: Glucosinolate turnover in Arabidopsis seeds

Kathrin Meier, Markus D. Ehbrecht, and Ute Wittstock*

*** Correspondence:** Ute Wittstock, u.wittstock@tu-bs.de

Supplementary Figures

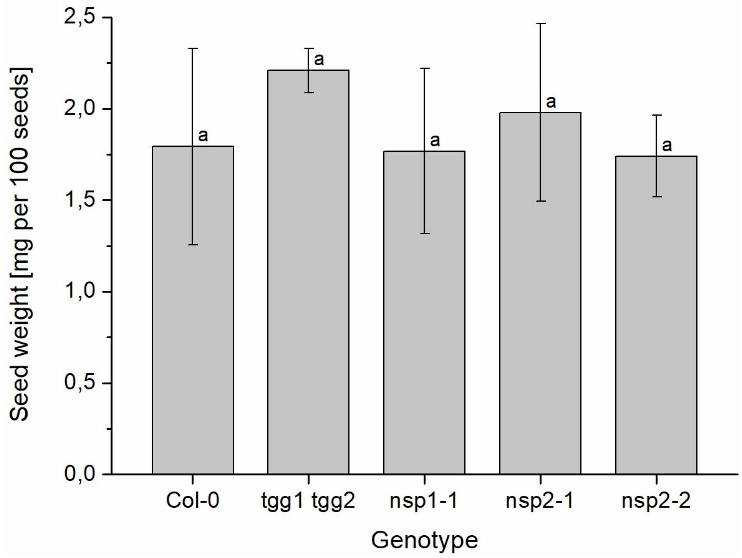

Fig. S1. Seed weight of the *A. thaliana* genotypes used in the study. A known number of seeds (100-200) was weighted on an analytical balance. The measured weight was used to calculate the weight of 100 seeds. Means ± SD of N=5-6 biological replicates. The same letter above bars indicates that there was no significant difference between genotypes ($p<0.05$, ANOVA).

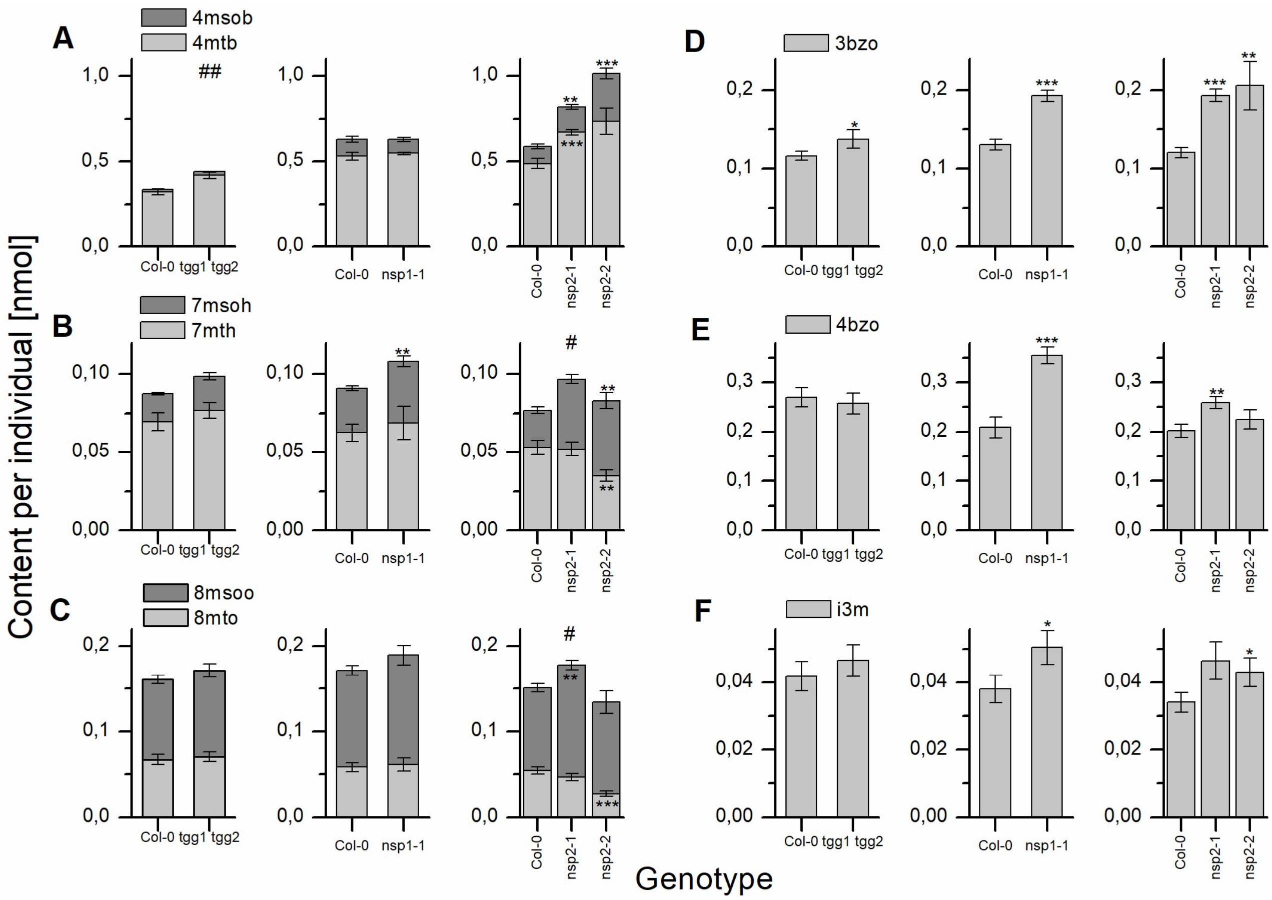

Fig. S2. Content of individual glucosinolates in dry seeds of *A. thaliana* Col-0 and breakdown pathway mutants. Total glucosinolate content was determined as nmol per individual. **A-C** depict methylthioalkylglucosinolates together with the derived methylsulfinylalkyl derivatives. **D-E** depict individual benzoylated glucosinolates and **F** indol-3-ylmethylglucosinolate. The left graph of each panel shows results of experiments with *tgg1 tgg2* in comparison with Col-0, the middle graph those with *nsp1* and Col-0, and the right graph those with two independent *nsp2* lines in comparison with Col-0. Means ± SD from N=3 biological replicates. Significant differences were identified by pairwise comparisons using Two-Sample-t-Test (if normal distribution was confirmed) or Mann-Whitney Test (if normal distribution could not be assumed). Asterisks indicate significant differences for individual glucosinolates relative to Col-0 (*, p<0.05; **, p<0.01; *** p<0.001) while hashs on top of a bar in **A-C** indicate significant differences for the sum of a biosynthetically linked pair relative to wildtype (#, p<0.05; ##, p<0.01).

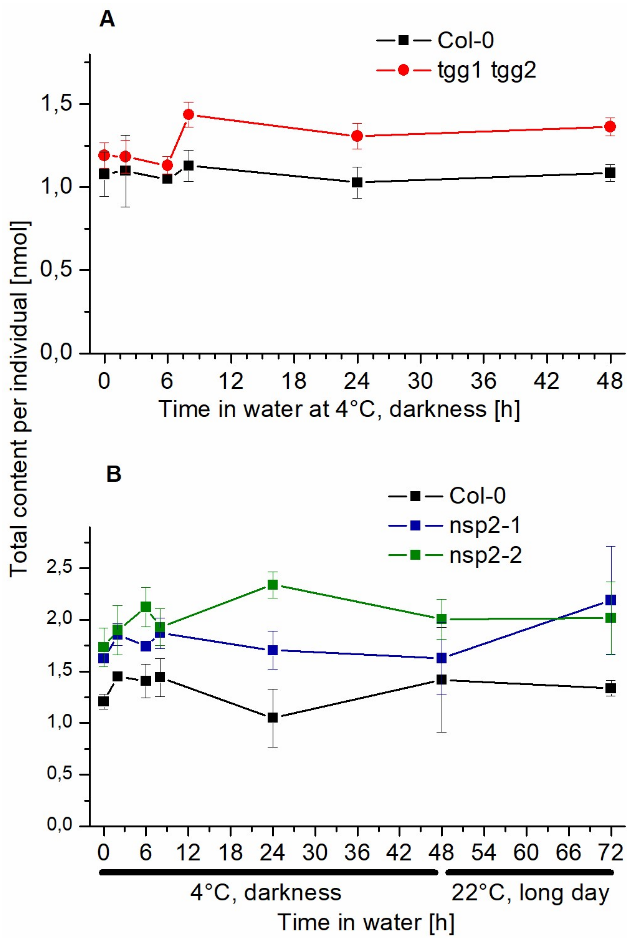

Fig. S3. Content of individual glucosinolates during 48 h stratification and 24 h of germination. Seeds were incubated in autoclaved tap water at 4°C in the dark for 48 h and (in case of nsp2 lines) moved to 22°C and 16 h photoperiod for another 24 h. Glucosinolate content is expressed as nmol per individual. **A**: Experiments with *tgg1 tgg2* in comparison with Col-0, **B**: Experiments with two independent *nsp2* lines in comparison with Col-0. Means ± SD from N=3 biological replicates (Col-0, 48 h in A, N=2).

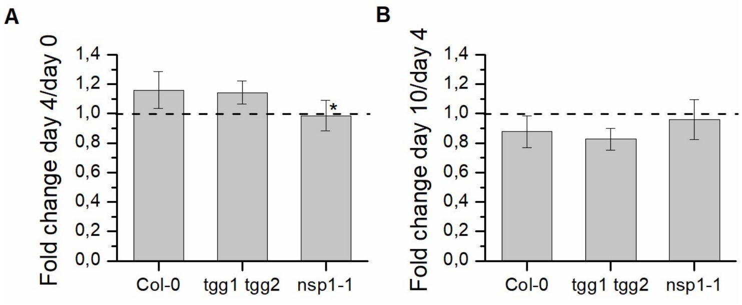

Fig. S4. Changes of seed glucosinolate content during germination on MS medium. Seeds of Col-0, *tgg1 tgg2* and *nsp1* were sterilized (day 0), plated on MS medium, stratified at 4°C in the dark for two days and then grown at 22°C with 16 h photoperiod. Total glucosinolate content was determined as nmol per individual. Fold change from day 0 to day 4 (**A**) and from day 4 to day 10 (**B**) is given. Ratio of 1 (no change) is highlighted by the dashed line. Means ± SD of N=7-9 biological replicates. Significant differences between mutant and Col-0 are indicated by asterisks (*, $p<0.05$, Two-Sample-t-Test).

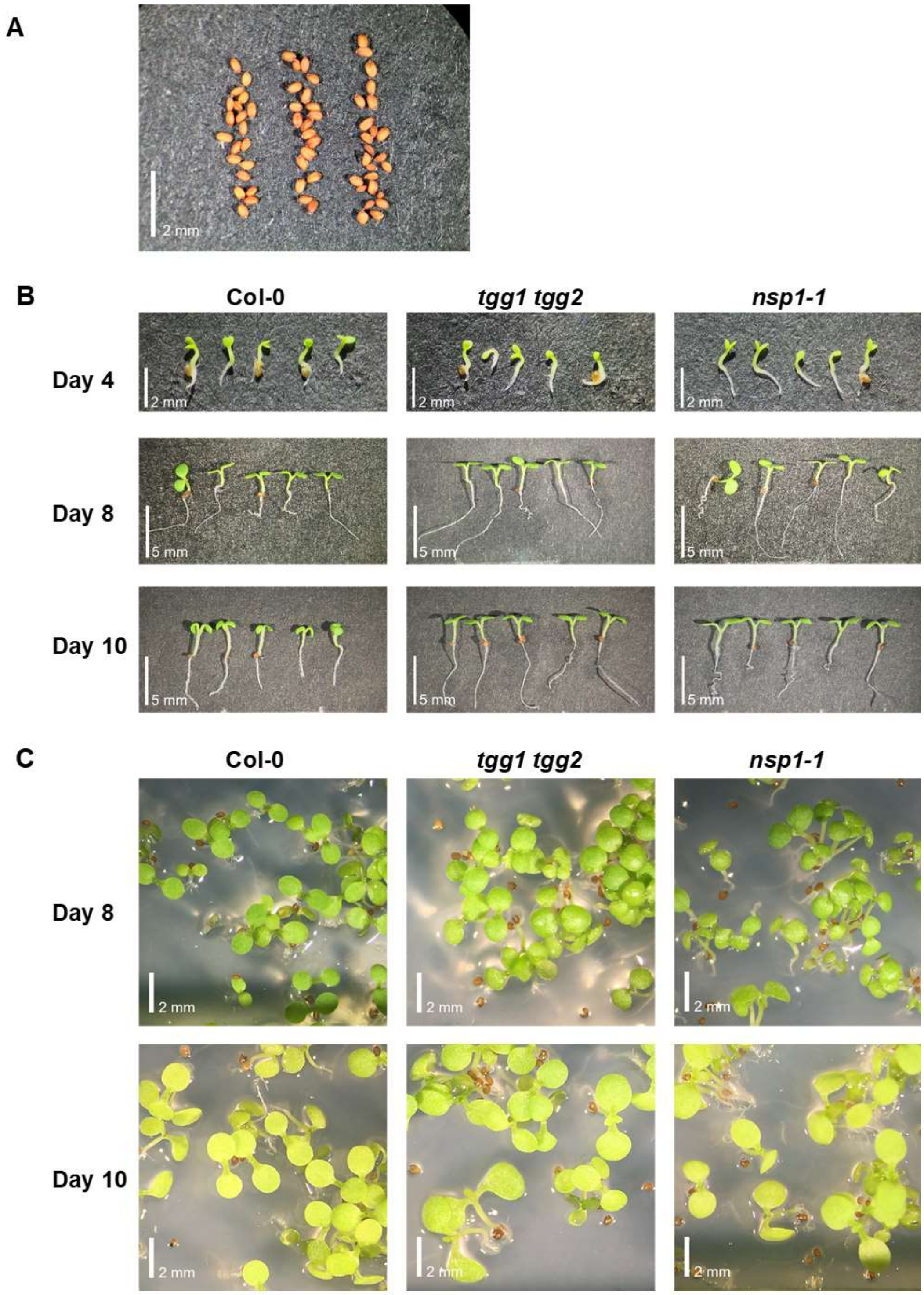

Fig. S5. Morphology of *A. thaliana* Col-0 in comparison to *tgg1 tgg2* and *nsp1-1* during seed-seedling transition. A. Dry seeds of Col-0 (left), *tgg1 tgg2* (middle), and *nsp1-1* (right). **B, C**. Seeds were sterilized (day 0), plated on MS medium, stratified at 4°C in the dark for two days and then grown at 22°C with 16 h photoperiod. Side view (**B**) and top view (**C**) of representative individuals.

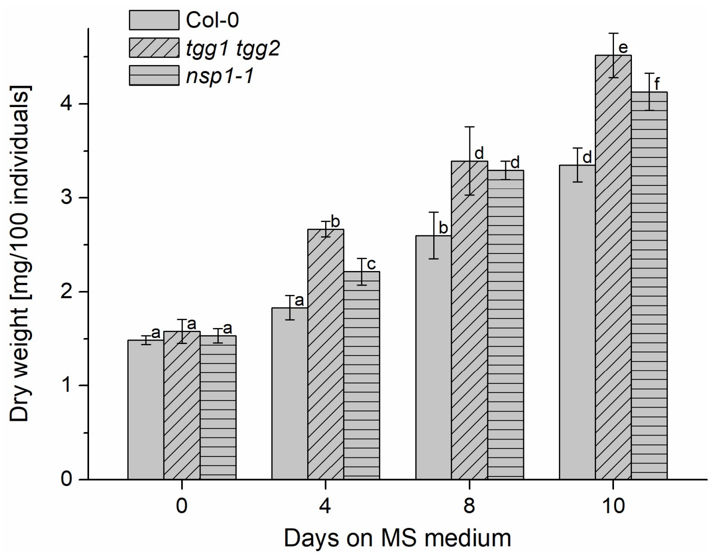

Fig. S6. Biomass gain of *A. thaliana* during seed-seedling transition. A known number of seeds (100-200) of Col-0, *tgg1 tgg2* and *nsp1* were sterilized (day 0), plated on MS medium, stratified at 4°C in the dark for two days and then grown at 22°C with 16 h photoperiod. Dry weight of pooled material from one plate was determined and used to calculate the weight of 100 individuals. Means ± SD of N=6 biological replicates. Different letters above the bars indicate significant differences (p<0.05, ANOVA with Tukey's test).

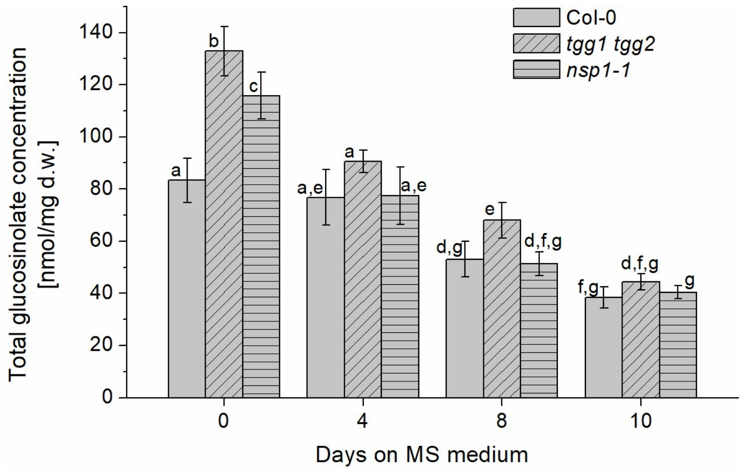

Fig. S7. Glucosinolate concentrations of *A. thaliana* during seed-seedling transition. A known number of seeds (100-200) of Col-0, *tgg1 tgg2* and *nsp1* were sterilized (day 0), plated on MS medium, stratified at 4°C in the dark for two days and then grown at 22°C with 16 h photoperiod. Glucosinolate content of pooled material from one plate was determined and related to dry weight. Means ± SD of N=6 biological replicates. Different letters above the bars indicate significant differences (p<0.05, ANOVA with Tukey's test).
